# Unveiling the Chemical Composition and Biological Properties of *Salvia cacaliifolia* Benth. Essential Oil

**DOI:** 10.3390/plants12020359

**Published:** 2023-01-12

**Authors:** Jorge M. Alves-Silva, Emma Cocco, Alessandra Piras, Maria José Gonçalves, Ana Silva, Danilo Falconieri, Silvia Porcedda, Maria Teresa Cruz, Andrea Maxia, Lígia Salgueiro

**Affiliations:** 1 Institute for Clinical and Biomedical Research, Health Sciences Campus, University of Coimbra, Azinhaga de S. Comba, 3000-548 Coimbra, Portugal; 2 Faculty of Pharmacy, Health Sciences Campus, University of Coimbra, Azinhaga de S. Comba, 3000-548 Coimbra, Portugal; 3 Laboratory of Plant Biology and Pharmaceutical Botany, Department of Life and Environmental Sciences, University of Cagliari, Viale Sant’Ignazio, 09123 Cagliari, Italy; 4 Department of Chemical and Geological Sciences, University of Cagliari, Cittadella Universitaria, 09042 Monserrato, Italy; 5 Chemical Process Engineering and Forest Products Research Centre, Department of Chemical Engineering, Faculty of Sciences and Technology, University of Coimbra, 3030-790 Coimbra, Portugal; 6 Center for Neuroscience and Cell Biology, Faculty of Medicine, University of Coimbra, Rua Larga, 3004-504 Coimbra, Portugal

**Keywords:** antifungal, anti-inflammatory, senescence, wound healing, sage

## Abstract

*Salvia* is widely recognized for its therapeutic potential. However, the biological relevance of some species remains unknown, namely *Salvia cacaliifolia* Benth. Therefore, the aim of this study is to unveil the chemical composition and relevant properties to its essential oil (EO). The EO was characterized by GC and GC-MS and its antifungal effect was evaluated according to the CLSI guidelines on dermatophytes and yeasts. The anti-inflammatory potential was assessed on lipopolysaccharide-stimulated macrophages, by assessing the production of nitric oxide (NO) and the effect on the protein levels of two key pro-inflammatory enzymes, iNOS and COX-2 by western blot analysis. Wound healing capacity was determined using the scratch wound healing assay, and the anti-aging potential was assessed by evaluating the senescence marker β-galactosidase. The EO was mainly characterized by γ-curcumene, β-bisabolene, bicyclogermacrene and curzerenone. It is effective in inhibiting the growth of dermatophytes and *C. neoformans*. The EO significantly decreased iNOS and COX-2 protein levels and concomitantly reduced NO release. Additionally, it demonstrated anti-senescence potential and promoted wound healing. Overall, this study highlights relevant pharmacological properties of the EO of *Salvia cacaliifolia*, which should be further explored envisaging the development of sustainable, innovative, and environmentally friendly skin products.

## 1. Introduction

Fungal infections remain neglected by public health authorities, affecting over a billion people with 1.5 million deaths per year [1]. Immunocompromised individuals are more prone to fungal infections [1], being *Candida* and *Cryptococcus* two main etiological agents for invasive infections [1,2]. Dermatophytes, another group of pathogenic fungi that are highly relevant due to their role in the morbidity-associated with superficial mycoses [3], includes *Trichophyton*, *Epidermophyton* and *Microsporum* strains. Furthermore, they can cause invasive diseases in immunocompromised hosts [4].

Dermatophytes can activate the immune system due to the binding of fungal epitopes, such as mannan [5,6], to toll-like receptors (TLRs) thus activating the pro-inflammatory cascade associated with these infections. One of such cascades is the nuclear factor kappa B (NF-κB) pathway, which ultimately leads to an increase in the production of inflammatory cytokines and the protein levels of key pro-inflammatory enzymes, such as inducible nitric oxide synthase (iNOS) and cycloxygenase-2 (COX-2) [7]. The enzyme iNOS is responsible for the production of nitric oxide (NO), which is a well-known pro-inflammatory mediator overproduced in several chronic inflammatory-related diseases.

Furthermore, dermatophytes are associated with the formation of skin lesions [8]. Therefore, after the resolution of an infection, wound healing processes must occur in order to regenerate the damaged tissue. This process encompasses different phases, that must occur in the right order and at the correct times, otherwise the wound becomes chronic [9].

Due to relapses, resistances and antifungal side-effects, current antifungal treatments are many times inefficient with fungal infections remaining an unmet clinical need [10]. Strikingly, an increasing number of reports demonstrate that several fungal strains have already developed resistance to current antifungal agents [11,12], justifying the development of new antifungal approaches [13]. Furthermore, anti-inflammatory drugs are also reported to cause several adverse effects [14]. Having this in mind, therapeutic agents that could concomitantly treat fungal infections and decrease the associated inflammation would be of uttermost importance.

Aromatic plants and their metabolites have interesting organoleptic characteristics that also contribute to their high value in pharmaceutical, agronomic, food, sanitary, cosmetic, and perfume industries [15], and emerge as potential candidates as new anti-inflammatory/antifungal drugs. 

Many aromatic plants from the Asteraceae, Apiaceae and Lamiaceae families are rich in essential oils that have demonstrated a wide array of biological effects, namely anti-inflammatory and antifungal properties [16,17,18,19,20,21,22]. The genus *Salvia* L., one of the most important genera of the Lamiaceae family, is cultivated to be used as food spices or flavoring agents in cosmetics and perfumery [23]. Several species are used in traditional medicine to treat microbial infections, malaria, inflammation, and to disinfect homes after sickness [23]. *Salvia* species have attracted researchers for their biological properties, showing strong antibacterial, antifungal, anticancer, and anti-inflammatory effects, as well as for improvement of cognitive performance [24,25,26,27,28,29,30]. Although this genus is largely studied, some species remain unknown regarding their chemical composition and their medicinal interest, such as *Salvia cacaliifolia* Benth. (syn *Salvia atriplicifolia* Fernald, *Salvia hempsteadiana* S.F.Blake and *Salvia mendax* Epling). *S. cacaliifolia* is an erect herbaceous perennial shrub reaching 70 cm in height, with triangular leaves and large racemes of deep blue, 2-lipped flowers 2 cm in length [31]. This species grows in high altitude regions from Mexico, Guatemala, and Honduras.

This works aims to chemically characterize the essential oil of *S. cacaliifolia* (cacalia sage) and to disclose its antifungal/anti-inflammatory potential. Furthermore, the wound healing capacity of the essential oil was also studied. To further promote the interest in this species, the anti-senescence potential was also examined.

## 2. Results

### 2.1. Chemical Composition of S. cacaliifolia Essential Oil

The essential oil was characterized by high amounts of hydrocarbons sesquiterpenes (70.10% ± 0.119) followed by oxygenated sesquiterpenes (11.71% ± 0.032), as shown in Table 1. Regarding individual compounds, the essential oil was rich in γ-curcumene (27.64% ± 0.195), β-bisabolene (18.01% ± 0.033), bicyclogermacrene (8.54% ± 0.074) and curzerenone (7.68% ± 0.055).

### 2.2. Antifungal Effect of Salvia cacaliifolia

The antifungal activity of the essential oil from *S. cacaliifolia*, particularly against dermatophytes and yeasts, was assessed. As shown in Table 2, the essential oil was effective against all tested dermatophyte strains, with *T. mentagrophytes* and *T. rubrum* being the most susceptible (MIC = 0.16 µL/mL). Regarding yeasts, *C. neoformans* was the most susceptible to essential oil (MIC = 0.64 µL/mL).

### 2.3. Anti-Inflammatory Potential of S. cacaliifolia 

**Figure 1:**
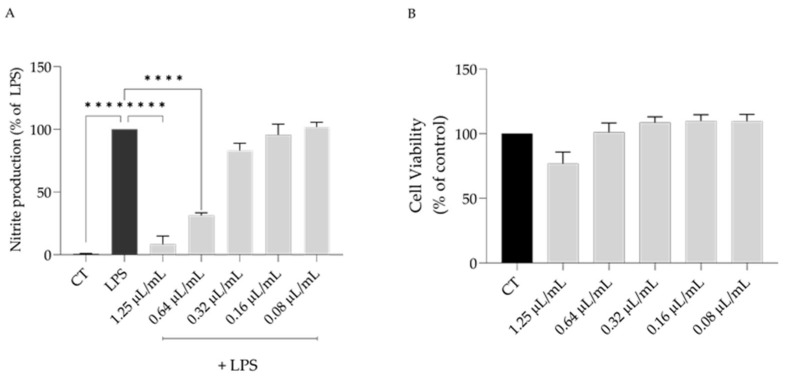
Effect of *S. cacaliifolia* essential oil on nitrite production (**A**) on LPS-stimulated macrophages after 1 h of pre-treatment with the essential oil followed by 24 h stimulation with LPS and on (**B**) cell viability after 24 h of cells treatment with the essential oil. The results show the mean ± SEM. **** *p* < 0.0001 when compared to control or LPS after one-way ANOVA followed by Dunnett’s multiple comparison test.

The anti-inflammatory potential of *S. cacaliifolia* essential oil was assessed using the lipopolysaccharide (LPS)-stimulated macrophages. As expected, the addition of LPS for 24 h lead to an increase in the secretion of nitric oxide (NO) to the culture medium ([NO] = 42.8 ± 7.4 µM). The addition of different concentrations of the essential oil led to a dose-dependent reduction in the nitrites detected (IC_50_ = 0.49 µL/mL). Both 1.25 and 0.64 µL/mL significantly decreased the detected nitrites (Figure 1A). Considering that at 1.25 µL/mL a significant decrease cell viability was observed (Figure 1B), the concentration of 0.64 µL/mL was used to disclose the underlying mechanism behind the anti-inflammatory potential of *S. cacaliifolia* essential oil.

**Figure 2:**
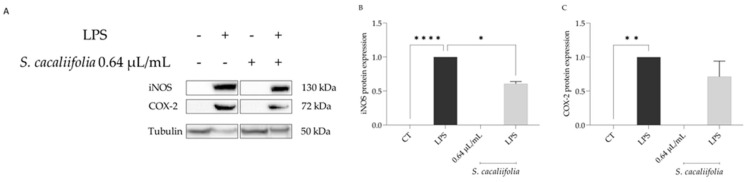
Effect of *S. cacaliifolia* essential oil on iNOS and COX-2 protein levels. (**A**) Representative blots of iNOS and COX-2 protein levels; + or − represents, respectively, the presence or absence of the condition in the table line. (**B**) Semi-quantitative analysis of iNOS protein levels. (**C**) Semi-quantitative analysis of COX-2 protein analysis. Protein levels were normalized to tubulin and to LPS. The results show the mean ± SEM. * *p* < 0.05, ** *p* < 0.01, **** *p* < 0.0001 when compared to control (CT) or LPS after one-way ANOVA followed by Dunnett’s multiple comparison test.

Considering that the binding of LPS to the Toll-like receptor 4 (TLR4) leads to the nuclear translocation of NF-κB which in turn causes the expression of inducible nitric oxide synthase (*Nos2*) and cycloxygenease-2 (*Cox2*) [34], we then assessed whether the presence of the essential oil could modulate the levels of both proteins. As shown in Figure 2A–C, the presence of LPS alone caused a significant increase in the protein levels of iNOS and COX-2. In the presence of the essential oil, both proteins’ levels were decreased, particularly that of iNOS (Figure 2A,B), suggesting that the oil impaired NF-κB activation, and consequently the expression of *Nos2* mRNA as well as its protein levels.

### 2.4. Wound Healing Properties of S. cacaliifolia Essential Oil

**Figure 3:**
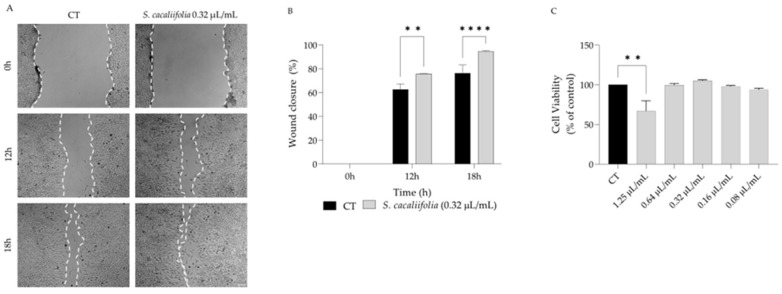
Effect of *S. cacaliifolia* on wound healing scratch assay. (**A**) Representative phase-contrast images of NIH 3T3 fibroblasts in the presence or absence of *S. cacaliifolia* essential oil (0.32 µL/mL) at 0 h, 12 h and 18 h post-scratch. (**B**) Quantification of the percentage of closed “wound”. (**C**) Effect of *S. cacaliifolia* essential oil on NIH 3T3 fibroblast viability. The results show the mean ± SEM. ** *p* < 0.01, **** *p* < 0.0001 when compared to control (CT) after two-way ANOVA followed by Sydák’s multiple comparison test or one-way ANOVA followed by Dunnett’s multiple comparison test.

Considering that several plants from the genus *Salvia* have been reported as wound healing agents [27,35,36], we hypothesise that *S. cacaliifolia* could promote wound healing. For that we resorted to the scratch assay and wound closure was assessed in the presence and absence of the essential oil in fibroblasts. We report that the essential oil significantly promoted the migration of fibroblasts when compared to control cells (Figure 3A,B) at 0.32 µL/mL, a concentration devoid of toxicity towards the fibroblasts (Figure 3C). 

### 2.5. Anti-Senescence Potential of S. cacaliifolia Essential Oil

Since it is known that some *Salvia* species present anti-senescence properties [37,38], we assessed the anti-senescence potential of *S. cacaliifolia* essential oil using the Senescence-associated β-galactosidase (β-gal) assay with etoposide as senescence-inducing agent. 

**Figure 4:**
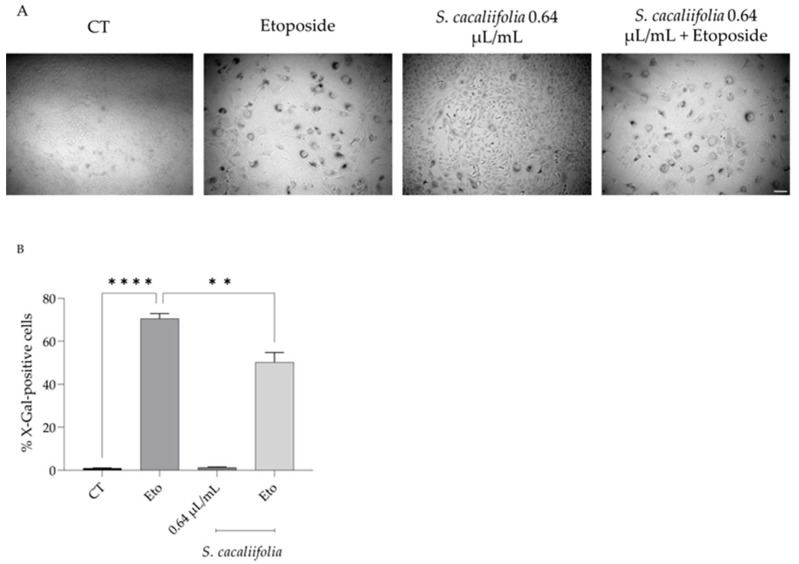
Effect of *S. cacaliifolia* essential oil on etoposide-induced cell senescence. (**A**) Representative phase-contrast images of NIH 3T3 fibroblasts treated with etoposide for 24 h followed by a recovery period of 72 h in the presence or absence of *S. cacaliifolia* essential oil. (**B**) Percentage of β-gal-positive cells. The results show the mean ± SEM. ** *p* < 0.01, **** *p* < 0.0001 when compared to control (CT) or etoposide (Eto) after one-way ANOVA followed by Dunnett’s multiple comparison test.

As shown in Figure 4, the addition of etoposide (12.5 µM) for 24 h followed by a recovery for 72 h in culture medium alone led to a significant increase in the β-gal-positive cells, showing that etoposide effectively induces cell senescence. Interestingly, when in the recovery phase *S. cacaliifolia* essential oil was added to the culture medium the percentage of β-gal-positive cells significantly decreased when compared to culture medium alone (Figure 4A,B), thus suggesting that *S. cacaliifolia* could also present anti-senescence effects.

## 3. Discussion

*S. cacaliifolia* is still largely unknown regarding the chemical composition of its essential oil as well as to any biologically relevant activities. To fill the gap in the knowledge, this work describes, for the first time, anti-inflammatory, anti-fungal, anti-senescence and wound healing properties for this species. 

We reported that *S. cacaliifolia* essential oil is predominantly characterized by sesquiterpenes, particularly γ-curcumene, β-bisabolene, bicyclogermacrene and curzerenone. Similarly, the essential oils from *S. scabra* and *S. aurea* were also rich in sesquiterpenes (92.4 and 75%, respectively) [39], with β-caryophyllene, epi-α-cadinol, δ-cadinene being the major compounds in *S. aurea* while *S. scabra* was characterized by germacrene D, β-caryophyllene, germacrene B, and α-copaene. *S. ceratophylla*, *S. verbenaca*, *S. stenophylla*, *S. runcinata* and *S. repens* are other examples of *Salvia* essential oils predominantly rich in sesquiterpenes [40,41,42]. However, no essential oils from *Salvia* presents high amounts of γ-curcumene, β-bisabolene and curzerenone as the essential oil from *S. cacaliifolia*. Other *Salvia* species present a very distinct chemical composition, with predominance of monoterpenes, such as *S. officinalis* with high amounts of *cis*-thujone and camphor [43,44], or 1,8-cineole and camphor [45] and *S. rosmarinus* rich in 1,8-cineole, α-pinene, camphor [46].

Considering that some *Salvia* species have been reported as agents able to treat skin infections [36,47], we then aimed to assess if *S. cacaliifolia* oil could have potential in controlling fungal infection. The essential oil showed inhibitory effects against all dermatophyte strains and *Cryptococcus neoformans*, thus suggesting that it could be used to treat fungal infections, particularly dermatophytosis and cryptococcosis. The essential oil from other species such as *S. officinalis* have inhibitory effect against dermatophytes with MIC ranging from 0.63 to 2.5 µL/mL [43,45], thus showing a weaker activity than the one reported for *S. cacaliifolia*. Two essential oils from *S. multicaulis* presented a relevant anti-dermatophytic activity, particularly the chemotype rich in nerolidol [48]. Regarding the activity of the main compounds of *S. cacaliifolia*, no studies have been carried out. However, some studies have assessed the antifungal activity of essential oils rich in these compounds. Indeed, a study reported the activity of several essential oils from *Helichrysum microphyllum* subsp. *tyrrhenicum* and showed that those with highest content in γ-curcumene presented the best antifungal activity [49]. Other studies reported moderate antifungal activity for essential oils rich in β-bisabolene [50,51], bicyclogermacrene [51,52] and curzerenone [53]. Considering the weak antifungal of the previously reported essential oils, we hypothesize that the activity of *S. cacaliifolia* might be attributed to synergistic effect between all the compounds in the mixture. The mechanisms of action underlying the antifungal activity of *S. cacaliifolia* essential oil and isolated compounds are still unknown. However, considering their lipophilic nature we hypothesize that they might induce fungal growth inhibition by integrating into membrane structures which would increase permeability, promote the leakage of intracellular components and enzyme inactivation [15,54]. 

Considering that fungal infections lead to the activation of the immune system [55], the anti-inflammatory potential of *S. cacaliifolia* was also highlighted in the present study since a decrease in NO release was achieved when macrophages were pre-treated with the essential oil prior to LPS stimulation. In order to disclose the underlying mechanisms of the anti-inflammatory potential of *S. cacaliifolia*, the effect of the essential oil on the protein levels of iNOS and COX-2, two pro-inflammatory proteins associated with the TLR4/NF-κB signaling pathway, was assessed. A decrease in both protein levels was observed, thus suggesting that the decrease in NO release might be due to an inhibition of the NF-κB pathway. Indeed, one of the key molecular mechanisms that contributes to the perpetuation of chronic inflammation is the activation of NF-κB signaling cascade, which has emerged as the master regulator of inflammation and innate immune homeostasis. Activation of the transcriptional factor NF-kB causes induction of COX-2, iNOS and also aberrant expression of the inflammatory cytokines tumor necrosis factor (TNF)-α, IL-1β, IL-6, which can damage healthy neighboring cells and over a long period of time may lead to chronic illnesses. Further studies need to be conducted in order to further understand the full mechanism behind the anti-inflammatory potential of *S. cacaliifolia* essential oil, such as the nuclear translocation of p65. Regarding other *Salvia* species, it was shown that *S. officinalis* essential oil at 0.64 µL/mL was able to decrease NO production by 30–40% [45], whereas the essential oil from *S. cacaliifolia* at the same dose decreases NO production by 70%. In opposition, the essential oil from *S. ceratophylla* greatly inhibits NO production (IC_50_ = 90 µg/mL) [40]. The capacity of *Salvia* species to modulate NF-kB pathway is already reported. Indeed, *S. officinalis* was able to decrease the expression of NF-kB [44]. *S. ceratophylla* strongly inhibits NF-κB with an IC_50_ of 38 µg/mL [40]. It was shown that *Helichrysum italicum* essential oil rich in γ-curcumene, the main compound of *S. cacaliifolia* oil, was able to decrease NO production, which was dependent on the content in this compound, which was corroborated by fractioning of the essential oil where the curcumene-rich fraction induced the highest inhibition [56]. Similar effects were described for the γ-curcumene chemotype of *H. italicum* [57]. It was shown that essential oils with significant amounts of β-bisabolene [58] and bicyclogermacrene [59] exerted promising anti-inflammatory effects. Regarding curzerenone, no studies have been conducted assessing its anti-inflammatory potential. However, it was reported that two essential oils rich in curzerenone have relevant antioxidant properties [60,61], which could also contribute to the anti-inflammatory activity of *S. cacaliifolia* by allowing NO scavenging. Having this in mind, it is possible that γ-curcumene, the main compound of the essential oil, might be the major player responsible for *S. cacaliifolia* anti-inflammatory activity, however the contribution of the remaining major compounds cannot be discarded.

Considering that dermatophyte infection can lead to the formation of skin lesions [8], we assessed whether *S. cacaliifolia* could promote wound healing. Our results showed that this essential oil promotes the migration of fibroblasts, thus suggesting that it might play a role in the wound healing process. An essential oil from *H. italicum* rich in γ-curcumene promoted wound healing in diabetic rats [62], therefore we hypothesize that the wound healing capacity of *S. cacaliifolia* might be attributed to the high amount of this compound.

Considering that several *Salvia* species can act as anti-senescence agents [37], we aimed to disclose if *S. cacaliifolia* could also revert senescence. Indeed, a reduction in the activity of senescence-induced pH-dependent β-galactosidase was observed. This activity might be attributed mainly to the presence of β-bisabolene since an essential oil rich in this compound also presented a strong anti-senescence effect on fibroblasts [63].

## 4. Conclusions

This work highlights the chemical composition of a *Salvia* species, that remains largely unknown, and demonstrates that this plant possesses several biologically relevant activities. The essential oil from *S. cacaliifolia* presents a distinct chemical composition, being predominantly rich in γ-curcumene, β-bisabolene, bicyclogermacrene and curzerenone. Furthermore, this is the first report showing the antifungal, anti-inflammatory, anti-senescence and healing properties of the *S. cacaliifolia* essential oil, suggesting its potential use as an ingredient for the development of cosmetic products for skin inflammageing prevention, as well as for the treatment of dermatophytosis and associated inflammation.

## 5. Materials and Methods

### 5.1. Plant Material

*Salvia cacaliifolia* seedlings have been grown from seed in “Planta Medica” greenhouse in the Laboratory of Plant Biology and Pharmaceutical Botany of the University of Cagliari (UNICA). Cacalia sage seeds, from Mexican origin, have been purchased from an Italian specialist store (http://www.leessenzedilea.com/). After 5 weeks, seedlings were transplanted to “Planta Medica” greenhouse in accordance with the eco-physiological needs of species. After 2 years of growth, the plants were harvested (blooming period) and dried in a laboratory for two days. Voucher specimen (6/23.9/V1) was deposited in the Herbarium Karalitanum (CAG), University of Cagliari, Viale S. Ignazio, 13 Cagliari, Italy.

### 5.2. Essential Oil Analysis

Isolation of essential oils by hydrodistillation were performed in a Clevenger-type apparatus for 3 h [64].

Analyses of the oils were carried out by both gas chromatography (GC) and gas chromatography/mass spectrometry (GC/MS). GC analyses were performed using a gas chromatograph (Agilent 7890A, Palo Alto, CA, USA), equipped with a 30 m × 0.25 mm i.d. with 0.25 µm stationary film thickness HP-5 capillary column (Agilent J&W, Santa Clara, CA, USA). The following temperature program was used: from 60 °C to 246 °C at a rate of 3 °C min^−1^ and then held at 246 °C for 20 min (total analysis time 82 min). Other operating conditions were the following: carrier gas helium (purity ≥ 99.9999%—Air Liquide, Milan, Italy); flow rate, 1.0 mL.min^−1^; injector temperature, 250 °C; detector temperature, 300 °C. Injection of 1 μL of diluted sample (1:100 in n-hexane, *w*/*w*) was performed with 1:20 split ratio, using an autosampler (Agilent, Model 7683B, Santa Clara, CA, USA).

GC-MS analyses were carried out using a gas chromatograph (Agilent 6890N, Santa Clara, CA, USA) equipped with a 30 m × 0.25 mm i.d. with 0.25 µm stationary film thickness HP-5ms capillary column (Agilent J&W, Santa Clara, CA, USA) coupled with a mass selective detector having an electron ionization device, EI, and a quadrupole analyzer (Agilent 5973, Santa Clara, CA, USA). The temperature program and the chromatographic operating conditions (except detector) were the same used for GC-FID. The MS conditions were as follows: MS transfer line temperature 240 °C; EI ion source temperature, 200 °C with ionization energy of 70 eV; quadrupole temperature 150 °C; scan rate, 3.2 scan.s^−1^ at m/z scan range, (30 to 480). To handle and process chromatograms and mass spectra was used the software MSD ChemStation (Agilent, rev. E.01.00.237, Santa Clara, CA, USA). Compounds were identified by comparison of their mass spectra with those of NIST02 library data of the GC/MS system and Adams libraries [32,33]. The results were further confirmed by comparison with the compounds elution order with their retention indices on semi-polar phases reported in the literature [32]. Retention indices of the components were determined relative to the retention times of a series of n-alkanes (two standard mix C8–C20 and C21–C40) with linear interpolation [65]. Percentage of individual components was calculated based on GC peak areas without FID response factor correction. The results are shown as the % of individual peaks ± standard deviation of two independent chromatographic runs.

### 5.3. Antifungal Activity

#### 5.3.1. Fungal Strains

The antifungal activity of the essential oil of *S. cacaliifolia* was evaluated against filamentous fungi and yeasts. Three dermatophyte clinical strains isolated from nails and skin (*Epidermophyton floccosum* FF9, *Trichophyton mentagrophytes* FF7 and *Microsporum canis* FF1), and four dermatophyte type strains from the Colección Espanõla de Cultivos Tipo (*T. mentagrophytes* var. *interdigitale* CECT 2958, *T. rubrum* CECT 2794, *T. verrucosum* CECT 2992, and *M. gypseum* CECT 2908), one *Cryptococcus neoformans* type strain (*C. neoformans* YPO186), two clinical *Candida* strain isolated from recurrent cases of vulvovaginal (*C. krusei* LF33, *C. guillermondii* MAT23) and three *Candida* type strains (*C. albicans* ATCC 10231, *C. tropicalis* YPO128 and *C. parapsilopsis* ATCC 90018). All strains were stored in Sabouraud dextrose broth with 20% glycerol at −80 °C and subcultured in Sabouraud dextrose agar (SDA) or Potato dextrose agar (PDA) before each test, to ensure optimal growth conditions and purity.

#### 5.3.2. Macrodilution Broth Assay

A macrodilution broth method was used to determine the minimal inhibitory concentrations (MIC) and the minimum lethal concentration (MLC) of the oil according to the Clinical and Laboratory Standards Institute (CLSI) reference protocol M38-A2 [66] or M27-A3 [67] for filamentous fungi and yeasts, respectively. The MIC was the lowest concentration in which no growth was observed in the inoculated test tubes, whereas the MLC was the lowest concentration where no growth was observed after inoculation in SDA of all the negative tubes. A negative (non-inoculated medium) and a positive (inoculated medium with 1% DMSO) controls were also included. 

### 5.4. Anti-Inflammatory Activity

#### 5.4.1. Cell Culture

RAW 264.7, a mouse leukemic macrophage cell line obtained from the American Type Culture Collection (ATCC TIB-71), was cultured as previously reported by our group [22].

#### 5.4.2. Nitric Oxide Production

NO production was measured by quantifying the accumulation of nitrites in culture supernatants, using the Griess reagent [68]. Cells (0.6 × 10^6^ cells/well) were cultured in 48-well culture plates. After an overnight stabilization, macrophages were pre-treated for 1 h with the essential oil (0.08–1.25 μL/mL) diluted in DMSO and then activated with 50 ng/mL of LPS during 24 h. Positive (LPS-stimulated macrophages) and negative controls (untreated macrophages) were performed. After this incubation period, equal volumes of culture supernatants and Griess reagent [1:1 of 0.1% (*w*/*v*) N-(1-naphthyl) ethylenediaminedihydrochloride and 1% (*w*/*v*) sulphanilamide containing 5% (*w*/*v*) H_3_PO_4_] were mixed and incubated for 30 min, in the dark. The absorbance at 550 nm was registered in an automated plate reader (Agilent, Santa Clara, CA, USA) and nitrite concentration was determined from a sodium nitrite standard curve. DMSO at the maximum concentration used (0.4%) was already demonstrated by our group to be devoid of anti-inflammatory and cytotoxicity effects (data not shown).

#### 5.4.3. Expression of Pro-Inflammatory Proteins, iNOS and COX-2

RAW 264.7 (1.2 × 10^6^ cells/well) were cultured in 6-well plates and allowed to stabilize for 12 h. Next, cells were incubated with the essential oil (0.64 µL/mL) for 1 h followed by LPS (50 ng/mL) activation during 24 h. A negative control (untreated cells) and a positive control (LPS only treated cells) was considered. Cell lysates were prepared as previously described by Zuzarte et al. [22].

Western blot analysis was carried out to measure the protein levels of inducible nitric oxide synthase (iNOS) and cyclooxygenase-2 (COX-2). The proteins were separated by electrophoresis on SDS-polyacrylamide 10% (*v*/*v*), at 130 V for 1.5 h, and transferred to polyvinylidene fluoride membranes (previously activated with methanol) at 400 mA for 3 h. After blocking non-specific IgGs with 5% (*w*/*v*) milk in TBS-T for 1 h at room temperature, membranes were incubated with specific antibodies against iNOS (1:500; R & D Systems) or COX-2 (1:5000; Abcam, Cambridge, UK) overnight, at 4 °C. Next, membranes were washed for 30 min with TBS-T (10 min, 3 times) and incubated at room temperature, for 1 h, with secondary antibodies (1:40,000; Santa Cruz Biotechnology, Dallas, TX, USA) conjugated with horseradish peroxidase. Immunocomplexes were detected using a chemiluminescence scanner (Image Quant LAS 500, GE, Boston, MA, USA). Membranes were probed with an anti-tubulin antibody (1:20,000; Sigma) to guarantee that an equivalent amount of protein was loaded. Protein quantification was performed using ImageLab version 6.1.0 (Bio-Rad Laboratories Inc., Hercules, CA, USA).

### 5.5. Cell Migration

#### 5.5.1. Cell Culture

NIH 3T3, a mouse embryonic fibroblast cell line (ATCC CRL-1658), was cultured as previously described in our group [69]. 

#### 5.5.2. Cell Migration Assay

Cell migration was carried out using the scratch wound assay as reported by Martinotti and colleagues [70] with slight modifications. Briefly, NIH 3T3 fibroblasts were seeded at 2.5 × 10^5^ cells/mL in 12-well plates. After 24 h of growth, a scratch was carried out in the cell monolayer using a 20–200 µL pipette tip. Detached cells were removed by washing cells with sterile PBS 1x. DMEM with 2% serum was added to all plates, in the presence or absence of the essential oil. Using phase-contract microscope, images were acquired 0, 12 and 18 h post-scratch, and the wound area was measured using ImageJ/Fiji software. The results presented were obtained using the following equation

$$wound\;closure\;\left(\%\right)=\frac{A_{t=0h}-A_{t=xh}}{A_{t=0h}}\times 100$$

where *A_t_*_=0*h*_ is the area of the wound 0 h after the scratch and *A_t_*_=*xh*_ is the area at the different time post-scratch (0 h, 12 h and 18 h).

### 5.6. Cell Viability 

The effect of different concentrations of the essential oil on the viability of both macrophages and fibroblasts was carried out using the resazurin reduction assay. Briefly, macrophages (0.6 × 10^6^ cells/mL), or fibroblasts (1.25 × 10^5^ cells/mL) were seeded in 48-well plates. After an overnight stabilization, different concentrations of the essential oil (0.08–1.25 μL/mL) diluted in DMSO were added for 24 h. At the end of the experiment, the medium was removed and fresh medium containing resazurin (1:10) was added for 1 h or 4 h, for macrophages and fibroblasts, respectively. The absorbance at 570 nm with a reference filter 620 nm was registered in an automated plate reader (Agilent, Santa Clara, CA, USA). Cell viability was determined using the following equation:

$$Cell\;viability\;\left(\%\right)=\frac{Abs_{Exp}}{Abs_{CT}}\times 100$$

where *Abs_Exp_* is the absorbance (difference between 570 and 620 nm) in the different experimental conditions and *Abs_CT_* is the absorbance in control cells (no essential oil).

### 5.7. Etoposide-Induced Senescence

Senescence was assessed using a commercially available beta-galactosidase staining kit according to the manufacturer’s protocol (Cell Signaling Technology). Briefly, 2.5 × 10^4^ fibroblasts were seeded in 12-well plates and allow for overnight adherence. Next, senescence was induced by incubating cells with 12.5 μM of etoposide for 24 h. Etoposide was removed, and cells were washed with PBS 1x. Next, the cells were allowed to recover for 72 h in DMEM in the absence or in the presence of *S. cacaliifolia* essential oil, and changes in morphology were assessed daily. After 72 h, cells were fixed for 15 min using 1x fixative solution (provided in the commercial kit), followed by PBS washes, and incubated overnight with beta-galactosidase staining solution in a dry incubator at 37 °C without CO_2_ supply. Different fields were viewed under microscope for blue color development and were photographed for image analysis (8 images per condition). A distinct blue color staining was indicative of beta-galactosidase activity. Quantitative analysis was carried out using ImageJ, and the percentage of senescent cells to total cells was calculated.

### 5.8. Statistical Analysis

The results are presented as mean values ± SEM (standard error of the mean) from at least three independent experiments performed in duplicate. Statistical significance for anti-inflammatory, cell viability and senescence assays were determined using one-way analysis of variance (ANOVA) followed by Dunnett’s post-hoc test using GraphPad Prism version 9.3.0 (GraphPad Software, San Diego, CA, USA). For cell migration assays statistical significance was determined by two-way ANOVA followed by Sydák’s multiple comparison test. A *p* value < 0.05 was considered to represent significant differences.

## Figures and Tables

**Table 1:** Chemical composition of *S. cacaliifolia* essential oil.

R_I_	Compound	*S. cacaliifolia* (% Peak Area, Mean ± SD)
1039	(Z)-β-Ocimene	0.24 ± 0.004
1050	(E)-β-Ocimene	1.02 ± 0.019
1375	α-Copaene	0.34 ± 0.004
1383	β-Bourbonene	0.22 ± 0.026
1390	β-Elemene	0.40 ± 0.002
1400	iso-Italicene	0.54 ± 0.005
1409	α-Cedrene	0.49 ± 0.021
1417	β-Cedrene	1.41 ± 0.008
1457	(E)-β-Farnesene	0.39 ± 0.009
1460	allo-Aromadendrene	3.02 ± 0.018
1462	cis-Cadina-1(6),4-diene	0.84 ± 0.005
1480	γ-Curcumene	27.64 ± 0.195
1483	Germacrene D	4.47 ± 0.043
1495	Bicyclogermacrene	8.54 ± 0.074
1510	β-Bisabolene	18.01 ± 0.033
1512	β-Curcumene	1.46 ± 0.060
1522	δ-Cadinene	0.68 ± 0.003
1537	α-Copaen-11-ol	0.68 ± 0.005
1555	Germacrene B	0.98 ± 0.014
1575	α-Cedrene epoxide	1.40 ± 0.018
1603	Curzerenone	7.68 ± 0.055
1652	Atractylone	0.68 ± 0.012
1683	α-Bisabolol	0.93 ± 0.016
1693	Germacrone	1.02 ± 0.014
Total identified		83.07 ± 0.112
Hydrocarbon monoterpenes		1.26 ± 0.024
Hydrocarbon sesquiterpenes		70.10 ± 0.119
Oxygenated sesquiterpenes		11.71 ± 0.032

R_I_—Retention index determined on a HP-5ms fused silica column relative to a series of n-alkanes. The results shown as mean ± SD of two independent analysis. Identification was achieved by comparing mass spectra (MS) to those found in NIST02 and Adams libraries [32,33] and retention indices (R_I_).

**Table 2:** Antifungal activity of *S. cacaliifolia* essential oil against dermatophytes and yeasts.

Strains	*S. cacaliifolia* Essential Oil	
	MIC ^a^	MLC ^a^
*Trichophyton mentagrophytes* FF7	0.16	0.16
*T. rubrum* CECT 2794	0.16	0.64
*T. mentagrophytes* var. *interdigitale* CECT 2958	0.32	0.32
*T. verrucosum* CECT 2992	1.25	2.5
*Microsporum canis* FF1	0.32	0.32
*M. gypseum* CECT 2908	0.32	1.25
*Epidermophyton floccosum* FF9	0.32	0.32
*Cryptococcus neoformans* YPO186	0.64	1.25
*Candida albicans* ATCC 10231	>2.5	>2.5
*C. tropicalis* YPO128	>2.5	>2.5
*C. krusei* LF33	>2.5	>2.5
*C. guillermondii* MAT23	2.5	>2.5
*C. parapsilosis* ATCC 90018	>2.5	>2.5

^a^ MIC and MLC were determined by a macrodilution method and expressed in μL/mL (*V*/*V*). The results were obtained from 3 independent experiments performed in duplicate.

## Data Availability

Data will be available upon request.
